# Classification of Early-Onset and Late-Onset Idiopathic Chronic Pancreatitis Needs Reconsideration

**DOI:** 10.1038/s41598-020-67306-w

**Published:** 2020-06-26

**Authors:** Yu Liu, Dan Wang, Yi-Li Cai, Tao Zhang, Hua-Liang Chen, Lu Hao, Teng Wang, Di Zhang, Huai-Yu Yang, Jia-Yi Ma, Juan Li, Ling-Ling Zhang, Cui Chen, Hong-Lei Guo, Ya-Wei Bi, Lei Xin, Xiang-Peng Zeng, Hui Chen, Ting Xie, Zhuan Liao, Zhi-Jie Cong, Zhao-Shen Li, Liang-Hao Hu

**Affiliations:** 1 Department of Gastroenterology, Changhai Hospital, The Second Military Medical University, Shanghai, 200433 China; 2 School of Basic medical sciences, The Second Military Medical University, Shanghai, 200433 China; 3 Department of Gastroenterology, First Affiliated Hospital, Zhejiang University School of Medicine, Hangzhou, 310006 China; 4 Department of Gastroenterology, Zhongda Hospital, Southeast University, Nanjing, 210000 China; 5 Department of General Surgery, Renji Hospital, Shanghai Jiaotong University, Shanghai, 200120 China

**Keywords:** Chronic pancreatitis, Gastroenterology

## Abstract

Bimodal classification of idiopathic chronic pancreatitis (ICP) into early-onset (<35 years) and late-onset (>35 years) ICP was proposed in 1994 based on a study of 66 patients. However, bimodal distribution wasn’t sufficiently demonstrated. Our objective was to examine the validity and relevance of the age-based bimodal classification of ICP. We analyzed the distribution of age at onset of ICP in our cohort of 1633 patients admitted to our center from January 2000 to December 2013. Classify ICP patients into early-onset ICP_(a)_ and late-onset ICP_(a)_ according to different cut-off values (cut-off value, a = 15, 25, 35, 45, 55, 65 years old) for age at onset. Compare clinical characteristics of early-onset ICP_(a)_ and late-onset ICP_(a)_. We found slightly right skewed distribution of age at onset for ICP in our cohort. There were differences between early-onset and late-onset ICP with respect to basic clinical characteristics and development of key clinical events regardless of the cut off age at onset i.e. 15, 25, 35, 45 or even higher. The validity of the bimodal classification of early-onset and late-onset ICP could not be established in our large patient cohort and therefore such a classification needs to be reconsidered.

## Introduction

Idiopathic chronic pancreatitis (ICP) has traditionally been defined as chronic pancreatitis (CP) in the absence of any obvious precipitating factors (e.g. alcohol abuse) and family history of the disease. In 1994, Layer *et al*. found distribution of the age at onset of ICP was bimodal^1^. According to bimodal phenomenon, they defined patients with age at onset of ICP < 35 years as early-onset ICP (EOICP) and those with age at onset of ICP > 35 years as late-onset ICP (LOICP)^1^.

Throughout these years, the classification was applied widely as a standard classification^2^. Several studies have found differences between EOICP and LOICP. It’s reported that EOICP patients have more severe pain^1,3^. Diabetes and pancreatic exocrine insufficiency were less frequent presenting symptoms in EOICP^1,3^. No significant difference was seen regarding pancreatic calcification between EOICP and LOICP^4^.

To our knowledge, no study has validated the distribution of age at onset of ICP after Layer *et al*. Bimodal distribution has never been shown by subsequent studies. For studies exhibiting age at onset of ICP, the distribution of age at onset of ICP was either not shown or shown as normal distribution (Table 1)^1,4–14^. Bimodal phenomenon was proposed based on a small sample study with only 66 ICP patients. Moreover, this distribution wasn’t statistically tested^1^. Thus, the distribution of age at onset of ICP remained to be explored.

**Table 1:** Previous studies about idiopathic chronic pancreatitis.

Items	Year	Mean age at onset±SD/SEM (Median age^*^)	Sample size, n	Country	distribution
Layer P^#1^	1994	—	66	USA	Bimodal distribution
Pfützer RH^7^	2000	16.4 ± 1.3	57	England	Normal distribution
Imoto M^8^	2000	41.68 ± NA	66	USA	Normal distribution
Bhatia E^6^	2002	19.7 ± 9.9	66	Germany	Normal distribution
Threadgold J^9^	2002	12 (7.5–20.5)^*^	108	UK	NA
Chandak GR^10^	2004	23.5 (22.8–27.3)^*^	120	India	NA
Chang MC^11^	2007	36.0 ± 17.1^*^	78	China	NA
Bhasin DK^4^	2009	30.6 ± 13.0	64	India	Normal distribution
Chang YT^5^	2009	43.0 ± NA	6	China	Normal distribution
Chang YT^5^	2009	27.0 ± NA	13	China	Normal distribution
Gasiorowska A^12^	2011	35 (17–56)^*^	14	Poland	NA
Midha S^13^	2010	24.69 ± 11.75	242	India	Normal distribution
Sun C^14^	2015	29 ± 15	58	China	Normal distribution
Sun C^14^	2015	38 ± 17	43	China	Normal distribution

ICP = idiopathic chronic pancreatitis, SD = standard deviation, SEM = standard error of mean.

^#^The “bimodal distribution” was proposed by Layer *et al*. While, we analyzed it was indeed uniform distribution.

^*^Median age (Quartile range)

Our objective was to correlate the age at onset of CP with disease course in order to examine the validity and relevance of the age-based bimodal classification of CP. In addition, we re-analyzed the age distribution of the study by Layer *et al*. which had suggested a bimodal age distribution.

## Materials and Methods

### Patients and database

Patients were from Changhai CP Database in which patients were retrospectively and prospectively enrolled. The detailed information of Changhai CP Database (version number 2.1, Shanghai, China) has been reported in our previous researches^15–22^. The exclusion criteria were as follows: pancreatic cancer diagnosed within 2 years after the diagnosis of CP^23^, groove pancreatitis^24^, autoimmune pancreatitis, and CP patients with distinct etiologies (including alcoholic, abnormal anatomy of pancreatic duct, hereditary, post-traumatic, and hyperlipidemic). All of the ICP patients in our database^16,18^ were enrolled in this study. All of the diagnostic modalities were carried out in accordance with the approved guidelines. All ICP patients were treated according to guidelines^25–27^.

The study was approved by the Ethics Committee of Changhai Hospital. Written informed consent was obtained from all participating patients. Patients or the public were not involved in the design, or conduct, or reporting, or dissemination of this research. All authors had access to the study data and reviewed and approved the final manuscript.

### Definitions of ICP and key clinical events in clinical course

Diagnosis of CP was established according to Asia-Pacific consensus^28^. The detailed diagnostic criteria of alcoholic CP, hereditary CP, post-traumatic CP, CP caused by hyperlipidemia, CP caused by abnormal anatomy of the pancreatic duct and ICP were described in our previous series reports^17,29^. The key clinical events of CP in clinical course included DM, steatorrhea, biliary stricture, pancreatic pseudocyst (PPC), pancreatic stones and pancreatic cancer. Diagnosis of DM was based on the criteria of the American Diabetes Association^30^. Diagnosis of steatorrhea was established when either of the following conditions was met: (1) chronic diarrhea with foul-smelling, oily bowel movements^31^; (2) a positive result in quantification of fecal fat determination (fecal fat quantification was performed over a period of three days; steatorrhea was defined as a fecal fat excretion of more than 14 g/day. Biliary stricture was defined as a narrowing of the biliary stricture with prestenosis dilation >1 cm on magnetic resonance imaging, computed tomography, or ultrasound, or delayed runoff of contrast on endoscopic retrograde cholangiopancreatography^32^. Diagnosis of PPC, pancreatic stones and pancreatic cancer was established according to guidelines^33–36^.

### Categories

CP with the age at onset of disease <35 years was defined as EOICP and those>35 was defined as LOICP according to Layer’s study^1^. To explore the uniqueness of Layer *et al*.’s classification, other cut-off values (a) for age at onset of ICP were selected as the new standard to classify EOICP_a_ and LOICP_a_ by increasing or decreasing the original cut-off value (35) by every 10 years. Thus, cut-off values, a (15, 25, 35, 45, 55, 65) were selected. Patients with the age at onset of ICP younger than the cut-off value were defined as EOICP_a_ and those with the age at onset of ICP older than the cut-off value were defined as LOICP_a._

### Statistical analysis

Continuous variables are expressed as the median (interquartile ranges) and were compared using an unpaired, 2-tailed *t* test for normally distributed data or the Mann-Whitney U test for non-normally distributed data. Categorical variables were compared using the χ^2^ test or the Fisher exact test. Kolmogorov–Smirnov test was used to identify description of age at onset of ICP via SPSS (version 23.0; SPSS, Chicago, IL).

Distribution of age at onset of ICP in Layer’s population and in our database was explored separately via Cullen and Frey graph drew from fitdistrplus package of R [version 3.5.0 (http://www.r-project.org/)] and Individual Distribution Identification together with Empirical Cumulative Distribution Function in Minitab software [version 18 (http://www.minitab.com/zh-cn/)]. The cumulative rates of key clinical events after the onset of ICP were calculated using the Kaplan-Meier method and differences between EOICP_a_ and LOICP_a_ were compared using Log-Rank test.

## Results

### Distribution of age at onset of ICP

**Figure 1:**
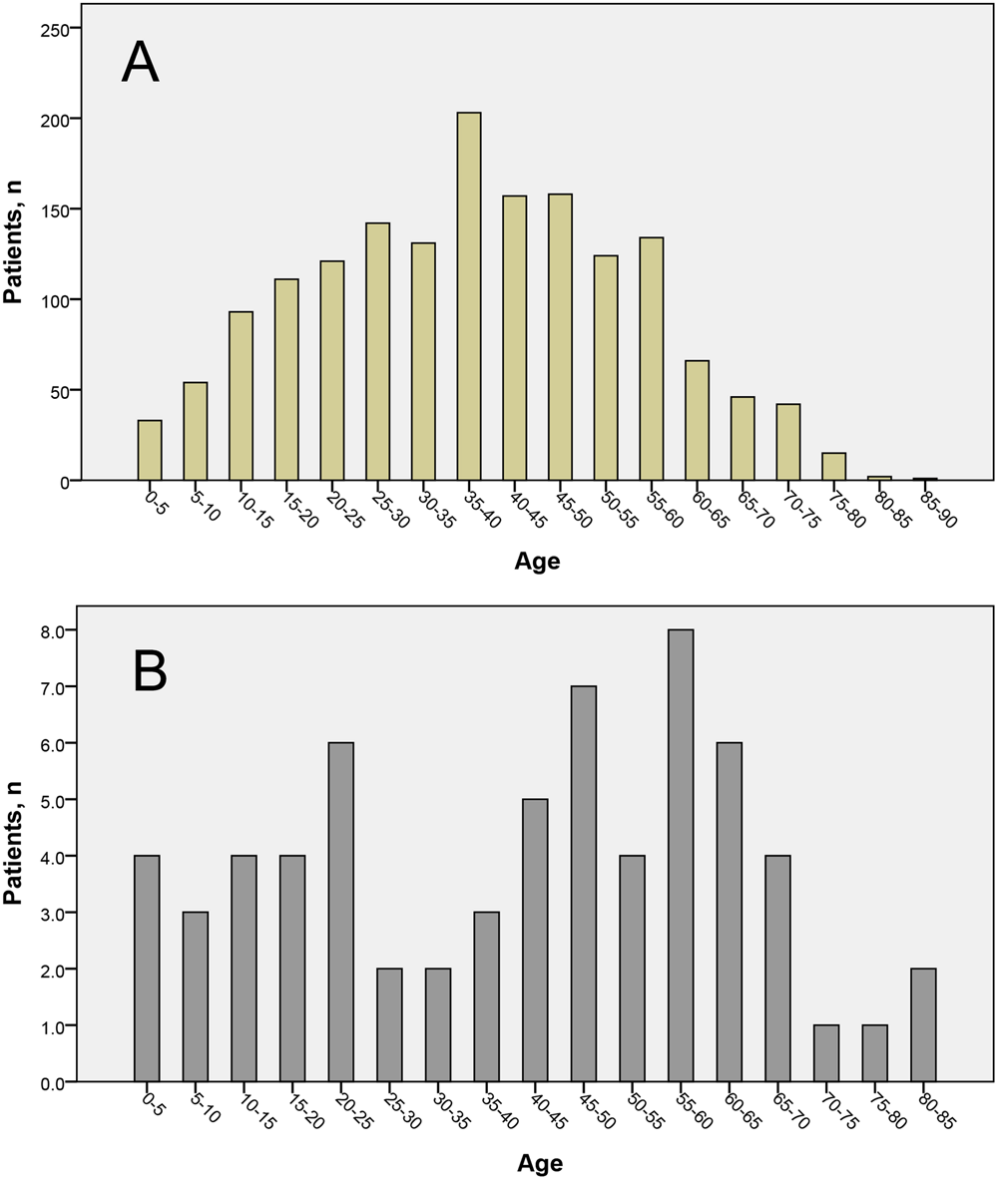
Distribution of age at onset of idiopathic chronic pancreatitis. (**A**) Distribution of age at onset of idiopathic chronic pancreatitis in our study. (**B**) Distribution of reconstructed data of age at onset of idiopathic chronic pancreatitis in Layer *et al*.’s study. ICP = idiopathic chronic pancreatitis.

Totally there were 1,633 ICP patients in our study and the baseline clinical and demographic data was exhibited in Supplementary Table 1. The median duration of follow-up was 9.8 (0.1, 53.2) years for all these 1,633 ICP patients. Bar graph for the distribution of age at onset of ICP was presented (Fig. 1A).

**Figure 2:**
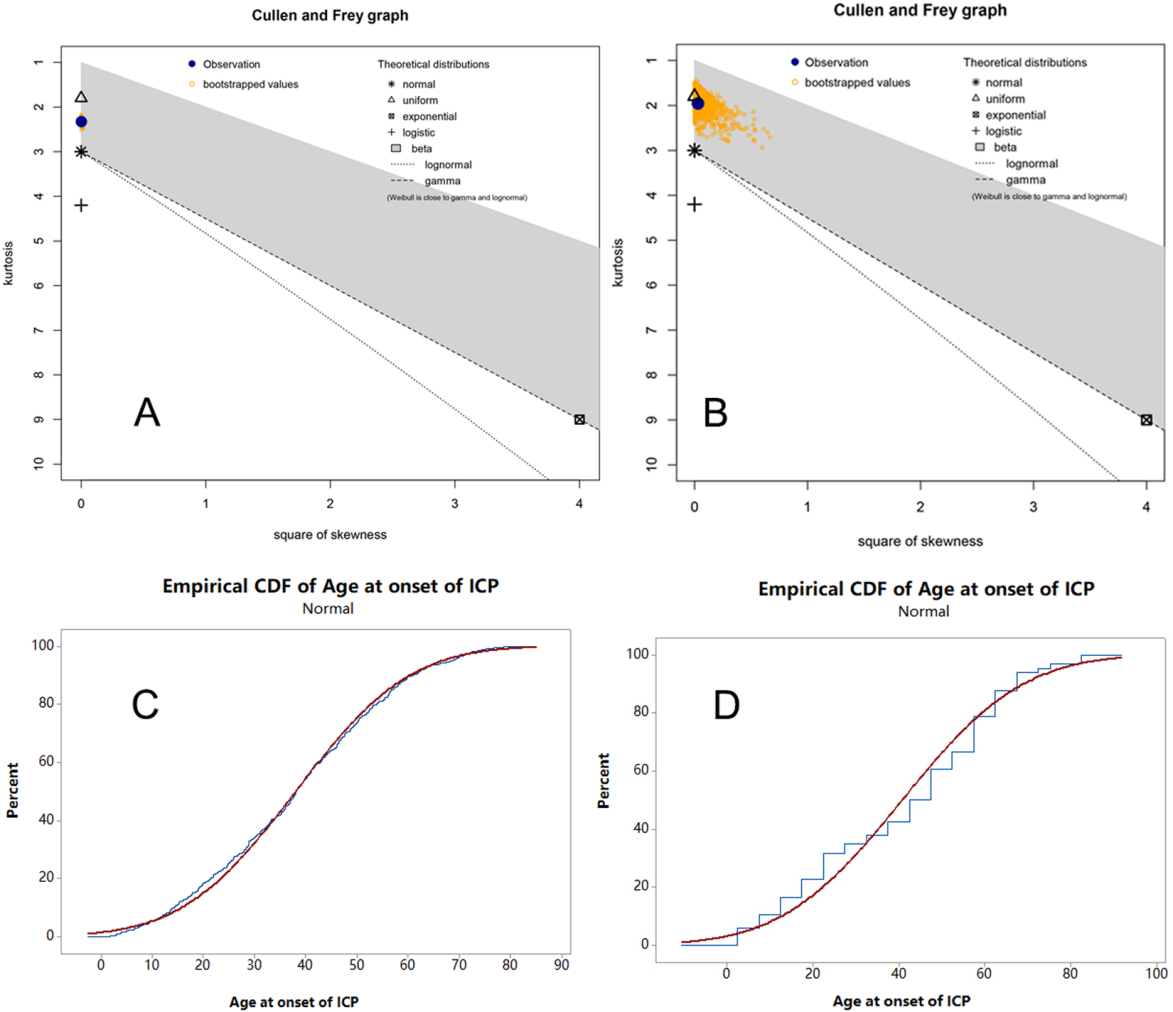
Distribution of age at onset of idiopathic chronic pancreatitis. (**A**) Cullen and Frey graph of distribution of age at onset of idiopathic chronic pancreatitis in our study. The blue dot (observation) was near the marker of normal distribution and uniform distribution but not overlaying the markers of them. (**B**) Cullen and Frey graph of distribution of age at onset of idiopathic chronic pancreatitis in Layer *et al*.’s study (calculated with reconstructed data). The blue dot (observation) was very close to the marker of uniform distribution. (**C**) Empirical cumulative distribution function for age at onset of idiopathic chronic pancreatitis in our study. (**D**) Empirical cumulative distribution function for age at onset of idiopathic chronic pancreatitis in Layer *et al*.’s study (calculated with reconstructed data). ICP = idiopathic chronic pancreatitis; CDF = empirical cumulative distribution function.

For ICP population in our study, Cullen and Frey graph revealed the distribution was not identical to uniform distribution or normal distribution (Fig. 2A). Further analysis showed there was right skewed distribution of the age at the onset of ICP with the skewness of 0.05 and the kurtosis of 2.33 calculated by R. The median age at onset of ICP was 38.21 years and mean age was 38.05 years (Supplementary Table 2). This skewed distribution was close to normal distribution which could be figured out through the empirical cumulative distribution function (Fig. 2C). Probability plot and empirical cumulative distribution function plot revealed there was no other satisfying distributions for age at onset of ICP in other thirteen classic distributions (Supplementary Figure 1, Supplementary Figure 2, Supplementary Table 3, Supplementary Table 4).

There were 66 ICP patients in Layer *et al*.’s study. The age at onset of ICP were reconstructed (Fig. 1B). Cullen and Frey graph revealed the distribution of Layer *et al*.’s population was nearly uniform distribution and it was far away from normal distribution (Fig. 2B). The empirical cumulative distribution function showed the distribution was not normal distribution (Fig. 2D). Kolmogorov–Smirnov test further verified that it was definitely uniform distribution (Supplementary Table 5, *P* = 0.224).

### General comparison of EOICP_a_ and LOICP_a_ with different cut-off values

When ICP patents were classified with distinct cut-offs, great differences were still observed between EOICP_a_ and LOICP_a_ (Table 2). Abdominal pain was dominant in initial manifestations and had higher proportion in EOICP_a_ than LOICP_a_ in the six classifications (*P* < 0.01). In the entire course of ICP, ratio of patients without pain was higher in LOICP_a_ than EOICP_a_ (when cut-off value equals 15, 25, 35, 45, 55: *P* < 0.001).

**Table 2:** Comparison of clinical course between early-onset idiopathic chronic pancreatitis_(a)_ and late-onset idiopathic chronic pancreatitis_(a)_ in different cut-offs of age at onset of idiopathic chronic pancreatitis.

Items	EOICP_15_ N = 180	LOICP_15_ N = 1453	EOICP_25_ N = 412	LOICP_25_ N = 1221	EOICP_35_ N = 685	LOICP_35_ N = 948	EOICP_45_ N = 1045	LOICP_45_ N = 588	EOICP_55_ N = 1327	LOICP_55_ N = 306	EOICP_65_ N = 1527	LOICP_65_ N = 106
Male sex	86 (47.8%)	945 (65.0%)^§^	216 (52.4%)	815 (66.7%)^§^	383 (55.9%)	648 (68.4%)^§^	642 (61.4%)	389 (66.2%)^#^	824 (62.1%)	207 (67.6%)^#^	959 (62.8%)	72 (67.9%)^#^
Age at the onset of CP, y*	10.401 (6.701, 13.019)	40.441 (29.319, 52.408)^§^	16.141 (11.256, 20.544)	44.030 (35.848, 55.123)^§^	22.022 (14.669, 28.788)	48.827 (40.990, 57.288)^§^	28.997 (18.944, 37.559)	55.458 (49.601, 62.158)^§^	33.619 (21.641, 43.219)	61.918 (57.651, 68.899)^§^	37.088 (23.915, 47.943)	70.610 (68.786, 73.358)^§^
Age at the diagnosis of CP, y*	16.380 (12.799, 22.129)	45.225 (35.595, 56.329)^§^	20.966 (16.362, 27.880)	48.269 (39.482, 58.143)^§^	28.266 (19.758, 34.840)	51.510 (44.251, 60.327)^§^	35.260 (24.311, 42.115)	58.169 (52.373, 63.864)^§^	38.885 (27.567, 47.904)	63.173 (59.526, 70.525)^§^	41.449 (29.770, 51.449)	72.343 (69.559, 75.422)^§^
Initial manifestations		^§^		^§^		^§^		^§^		^§^		^#^
Abdominal pain	176 (97.8%)	1170 (80.5%)	384 (93.2%)	962 (78.8%)	609 (88.9%)	737 (77.7%)	895 (85.6%)	451 (76.7%)	1125 (84.8%)	221 (72.2%)	1261 (82.6%)	85 (80.2%)
Endocrine/Exocrine dysfunction	4 (2.2%)	174 (12.0%)	18 (4.4%)	160 (13.1%)	49 (7.2%)	129 (13.6%)	106 (10.1%)	72 (12.2%)	138 (10.4%)	40 (13.1%)	168 (11.0%)	10 (9.4%)
Others	0 (0.0%)	109 (7.5%)	10 (2.4%)	99 (8.1%)	27 (3.9%)	82 (8.6%)	44 (4.2%)	65 (11.1%)	64 (4.8%)	45 (14.7%)	98 (6.4%)	11 (10.4%)
Pancreatic stones^†^	167 (92.8%)	1025 (70.5%)^§^	374 (90.8%)	818 (67.0%)^§^	596 (87.0%)	596 (62.9%)^§^	858 (82.1%)	334 (56.8%)^§^	1039 (78.3%)	153 (50.0%)^§^	1143 (74.9%)	49 (46.2%)^§^
Time between onset and pancreatic stone*	7.419 (2.499, 13.756)	2.255 (0.166,7.048)^§^	5.192 (1.419, 13.008)	2.003 (0.134, 6.332)^§^	4.430 (1.000, 11.112)	1.499 (0.061, 5.751)^§^	3.745 (0.832, 9.882)	0.666 (0.000, 4.836)^§^	3.255 (0.501, 9.006)	0.419 (0.000, 3.586)^§^	3.002 (0.337, 8.510)	0.022 (0.000, 2.436)^§^
DM	17 (9.4%)	413 (28.4%)^§^	52 (12.6%)	378 (31.0%)^§^	129 (18.8%)	301 (31.8%)^§^	251 (24.0%)	179 (30.4%)^||^	354 (26.7%)	76 (24.8%)^#^	405 (26.5%)	25 (23.6%)^#^
Time between onset and DM*	21.014 (2.252, 26.141)	1.416 (0.000, 7.170)^§^	16.011 (1.960, 23.012)	1.086 (0.000, 5.587)^§^	9.008 (0.373, 16.597)	0.753 (0.000, 3.822)^§^	3.323 (0.000, 10.589)	0.334 (0.000, 2.507)^§^	2.463 (0.000, 8.691)	0.000 (0.000, 0.979)^§^	2.003 (0.000, 7.838)	0.000 (0.000, 0.188)^§^
Steatorrhea	24 (13.3%)	315 (21.7%)^||^	72 (17.5%)	267 (21.9%)^#^	151 (22.0%)	188 (19.8%)^#^	239 (22.9%)	100 (17.0%)^||^	292 (22.0%)	47 (15.4%)^¶^	334 (21.9%)	5 (4.7%)^§^
Time between onset and steatorrhea*	12.595 (4.503, 21.014)	1.085 (0.000, 9.093)^§^	7.882 (1.416, 20.014)	1.000 (0.000, 8.005)^§^	5.005 (0.011, 13.841)	0.249 (0.000, 6.232)^§^	3.249 (0.000, 11.425)	0.162 (0.000, 5.175)^§^	2.463 (0.000, 10.443)	0.112 (0.000, 1.085)^§^	1.671 (0.000, 9.614)	6.838 (3.921, 8.501)^#^
Biliary stricture	14 (7.8%)	245 (16.9%)^||^	26 (6.3%)	233 (19.1%)^§^	49 (7.2%)	210 (22.2%)^§^	116 (11.1%)	143 (24.3%)^§^	172 (13.0%)	87 (28.4%)^§^	225 (14.7%)	34 (32.1%)^§^
Time between onset and CBD stenosis*	27.618 (12.258, 37.319)	1.501 (0.167, 6.145)^§^	21.016 (9.340, 31.628)	1.211 (0.167, 6.014)^§^	10.419 (4.332, 24.014)	0.916 (0.077, 4.444)^§^	4.332 (0.918, 10.982)	0.748 (0.077, 3.332)^§^	3.337 (0.427, 9.074)	0.299 (0.011, 2.137)^§^	2.167 (0.241, 8.188)	0.299 (0.127, 2.501)^||^
Pancreatic pseudocyst	17 (9.4%)	223 (15.3%)^#^	41 (10.0%)	199 (16.3%)^||^	89 (13.0%)	151 (15.9%)^#^	146 (14.0%)	94 (16.0%)^#^	186 (14.0%)	54 (17.6%)^#^	226 (14.8%)	14 (13.2%)^#^
Time between onset and pseudocyst formation*	2.836 (0.656, 8.838)	2.164 (0.290, 5.836)^#^	5.068 (1.238, 8.922)	1.608 (0.290, 5.249)^||^	4.737 (1.000, 8.496)	1.025 (0.290, 3.975)^§^	3.874 (0.331, 7.258)	1.000 (0.173, 3.605)^||^	3.501 (0.414, 7.258)	1.000 (0.052, 2.422)^§^	2.389 (0.329, 6.334)	1.000 (0.000, 2.558)^||^
Death	0 (0.0%)	57 (3.9%)^||^	4 (1.0%)	53 (4.3%)^§^	4 (0.6%)	53 (5.6%)^§^	12 (1.1%)	45 (7.7%)^§^	25 (1.9%)	32 (10.5%)^§^	38 (2.5%)	19 (17.9%)^§^
Pancreatic cancer	0 (0.0%)	18 (1.2%)^#^	2 (0.5%)	16 (1.3%)^#^	2 (0.3%)	16 (1.7%)^||^	7 (0.7%)	11 (1.9%)^¶^	14 (1.1%)	4 (1.3%)^#^	18 (1.2%)	0 (0.0%)^#^
Morphology of MPD‡		^§^		^§^		^§^		^§^		^§^		^§^
Pancreatic stone alone	67 (37.2%)	388 (26.7%)	137 (33.3%)	318 (26.0%)	217 (31.7%)	238 (25.1%)	303 (29.0%)	152 (25.9%)	379 (28.6%)	76 (24.8%)	433 (28.4%)	22 (20.8%)
MPD stenosis alone	38 (21.1%)	459 (31.6%)	79 (19.2%)	418 (34.2%)	144 (21.0%)	353 (37.2%)	253 (24.2%)	244 (41.5%)	355 (26.8%)	142 (46.4%)	442 (28.9%)	55 (51.9%)
MPD stenosis and stone	68 (37.8%)	451 (31.0%)	177 (43.0%)	342 (28.0%)	279 (40.7%)	240 (25.3%)	403 (38.6%)	116 (19.7%)	470 (35.4%)	49 (16.0%)	500 (32.7%)	19 (17.9%)
Complex pathologic changes	7 (3.9%)	155 (10.7%)	19 (4.6%)	143 (11.7%)	45 (6.6%)	117 (12.3%)	86 (8.2%)	76 (12.9%)	123 (9.3%)	39 (12.7)	152 (10.0%)	10 (9.4%)
Type of pain		^§^		^§^		^§^		^§^		^§^		^¶^
Recurrent acute pancreatitis	50 (27.8%)	437 (29.8%)	138 (33.5%)	349 (28.6%)	223 (32.6%)	264 (27.8%)	318 (30.4%)	169 (28.7%)	403 (30.4%)	84 (27.5%)	449 (29.4%)	38 (35.8%)
Recurrent pain	43 (23.9%)	471 (32.4%)	102 (24.8%)	412 (33.7%)	174 (25.4%)	340 (35.9%)	292 (27.9%)	222 (37.8%)	392 (29.5%)	122 (39.9%)	472 (30.9%)	42 (39.6%)
Recurrent acute pancreatitis and pain	75 (41.7%)	344 (23.7%)	144 (35.0%)	275 (22.5%)	223 (32.6%)	196 (20.7%)	317 (30.3%)	102 (17.3%)	381 (28.7%)	38 (12.4%)	406 (26.6%)	13 (12.3%)
Chronic pain	11 (6.1%)	68 (4.7%)	19 (4.6%)	60 (4.9%)	33 (4.8%)	46 (4.9%)	50 (4.8%)	29 (4.9%)	381 (28.7%)	38 (12.4%)	73 (4.8%)	6 (5.7%)
Without pain	1 (0.6%)	133 (9.2%)	9 (2.2%)	125 (10.2%)	32 (4.7%)	102 (10.8%)	68 (6.5%)	66 (11.2%)	85 (6.4%)	49 (16.0%)	127 (8.3%)	7 (6.6%)

CP = chronic pancreatitis, CBD = common biliary stricture, DM = diabetes mellitus, EOICP = early-onset idiopathic chronic pancreatitis, ICP = idiopathic chronic pancreatitis, LOICP = late-onset idiopathic chronic pancreatitis, MPD = main pancreatic duct, NS = nonsignificant.

*Median (interquartile ranges).

†Pancreatic calcifications were also regarded as stones that are located in branch pancreatic duct or ductulus.

‡MPD was classified as four types: pancreatic stone alone, MPD stenosis alone, MPD stenosis combined with stone and complex pathologic changes (patients with stricture, stones and also ductal dilatation in the body/tail area).

^§^Significant in comparison with EOICP_n-_ at P < 0.001.

^||^Significant in comparison with patients with EOICP_n-_ at P < 0.01.

^¶^Significant in comparison with patients with EOICP_n-_ at P < 0.05.

^#^Non-significant in comparison with patients with EOICP_n-_ at P > 0.05.

Pancreatic stones present higher proportion in EOICP_a_ than LOICP_a_ (*P* < 0.001). DM was found more frequently in LOICP_a_ than EOICP_a_ when cut-off value equals 15, 25, 35, 45 (*P* < 0.001), but the results were opposite when ICP patients were divided by 55 and 65 cutoffs with no significance. Steatorrhea was found more frequently in LOICP_a_ than EOICP_a_ when cut-off value equals 15 (*P* < 0.001) and 25 (*P* > 0.05) but the results were opposite when ICP patients were divided by 45, 55 and 65 cutoffs (all *P* < 0.05). Proportion of biliary stricture was higher in LOICP_a_ than EOICP_a_ (all *P* < 0.001). Proportion of PPC was higher in LOICP_a_ than EOICP_a_ (when cut-off value equals 15, 25, *P* < 0.05). Pancreatic cancer was more common in LOICP_a_ than EOICP_a_ when cut-off value equals 35 and 45 (*P* < 0.05). Death was more common in LOICP_a_ than EOICP_a_ in all the comparisons (*P* < 0.01).

### Comparisons of cumulative rates of key clinical events in EOICP_a_ and LOICP_a_

**Figure 3:**
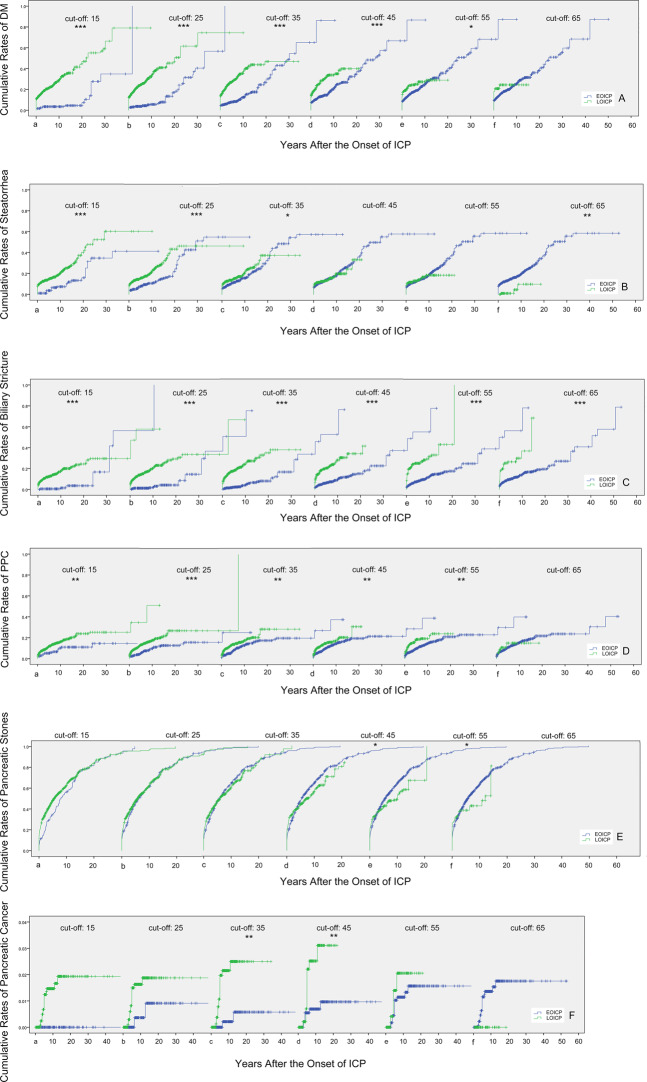
The cumulative rates after the onset of idiopathic chronic pancreatitis. (**A**) The cumulative rates of diabetes mellitus; (**B**) The cumulative rates of steatorrhea; (**C**) The cumulative rates of biliary stricture; (**D**) The cumulative rates of pancreatic pseudocyst; (**E**) The cumulative rates of pancreatic stone; (**F**) The cumulative rates of pancreatic cancer. The letter a, b, c, d, e and f refer to the zero point of the curve for different cut-off values. DM = diabetes mellitus, ICP = idiopathic chronic pancreatitis, PPC = pancreatic pseudocyst ***Significant in comparison of cumulative rates in early-onset idiopathic chronic pancreatitis_(a)_ (EOICP_a_) and late-onset idiopathic chronic pancreatitis_(a)_ (LOICP_a_) at *P* < 0.001. **Significant in comparison of cumulative rates in EOICP_a_ and LOICP_a_ at *P* < 0.01. *Significant in comparison of cumulative rates in EOICP_a_ and LOICP_a_ at *P* < 0.05. NS: No significance in comparison of cumulative rates in EOICP_a_ and LOICP_a_.

Significant difference in the cumulative rates of DM after the onset of ICP was observed between EOICP_a_ and LOICP_a_ patients when the cut-off values were 15, 25, 35, 45, 55 (*P* < 0.05, Fig. 3A). Significant difference in the cumulative rates of steatorrhea after the onset of ICP was observed between EOICP_a_ and LOICP_a_ patients when the cut-off values were 15, 25, 35, 65 (*P* < 0.05, Fig. 3B). Significant difference in the cumulative rates of biliary stricture after the onset of ICP was observed between EOICP_a_ and LOICP_a_ patients when the cut-off values were 15, 25, 35, 45, 55, 65 (*P* < 0.001, Fig. 3C). Significant difference in the cumulative rates of PPC after the onset of ICP was observed between EOICP_a_ and LOICP_a_ patients when the cut-off values were 15, 25, 35, 45, 65 (*P* < 0.01, Fig. 3D). Significant difference in the cumulative rates of pancreatic stones after the onset of ICP was observed between EOICP_a_ and LOICP_a_ patients when the cut-off values were 45, 65 (*P* < 0.05, Fig. 3E). Significant difference in the cumulative rates of pancreatic cancer after the onset of ICP was observed between EOICP_a_ and LOICP_a_ patients when the cut-off values were 35, 45 (*P* < 0.01, Fig. 3F).

## Discussion

To our previous knowledge, EOICP and LOICP were identified as two different entities due to the two distinct age groups of ICP patients. However, the bimodal distribution of age at onset of ICP was proposed based on a small sample and the distribution wasn’t statistically tested. Through our analysis, the distribution of reconstructed data for age at the onset of ICP patients in Layer *et al*.’s study turned out to be a uniform distribution rather than a bimodal distribution^1^. The existence of key clinical events and differences always exist no matter what the cut-off value is for EOICP_a_ and LOICP_a_. Therefore, the classification of EOICP and LOICP by Layer *et al*. needs to be reconsidered.

When classifying ICP patients with different cut-off values for the age at onset of ICP, cumulative rates of key clinical events after the onset of ICP were all different between EOICP_a_ and LOICP_a_ patients. Generally speaking, the development of key clinical events were more common in LOICP_a_ patients than EOICP_a_ patients except pancreatic stones. Similarly, 30 years old was selected as the cut-off value to classify EOICP_30_ and LOICP_30_ in an Indian study even though they didn’t mentioned why they chose 30 years old as the cut-off value, and similar differences in key clinical events between EOICP_30_ and LOICP_30_ were also found^3^. Thus, no matter which cut off value we use to classify EOICP_a_ and LOICP_a_, we always find the differences between EOICP_a_ and LOICP_a_. As a result, we assume that if the demarcation point value of the “bimodal distribution” of age at onset of ICP in Layer *et al*.’s study was just any other random number, EOICP and LOICP might have been defined by that random number in 1994.

Why do differences exist between EOICP_a_ and LOICP_a_? The advances in radiological imaging benefits the early diagnosis of ICP which shifted the diagnosis of LOICP group to a decade earlier^37^. It is possible that those identified as late onset have CP which is clinically silent for decades and present with exocrine and endocrine insufficiency at later age. These patients experienced less pain due to the long term chronic inflammation according to the pancreatic burnout theory^38^. It is possible there is a spectrum in which patients with intense inflammation present early and low grade chronic inflammation present late so that differences exist no matter what cut-off value was used to define EOICP and LOICP. From the perspective of gene, pancreatic secretory trypsin inhibitor (SPINK1) mutations have ever been considered to be the possible explanation of the difference between EOICP and LOICP because it was identified more frequent in EOICP than LOICP^39^, however, the association between the SPINK1 mutations and the age of onset of ICP is still pending. There is no definite range of age at onset of ICP in patients with SPINK1 mutations. Chang *et al*. just claimed SPINK1 mutation in ICP patients suggested earlier onset of age, higher frequency of constant pain, and earlier occurrence of pancreatic calcification and pseudocyst^5^. Sun *et al*. found the presence of the SPINK1 c.194 + 2 T > C mutation seemed to be associated with ICP and could predispose individuals to pancreatic diabetes at an earlier age^40^. However, Chandak *et al*. found that there was no statistical difference between age at onset of ICP patients with SPINK1 mutation and that of ICP patients without SPINK1 mutation^10^. Jalaly *et al*. concluded that ICP was not independently associated with pathogenic genetic variants^41^. The association of genetic factors and clinical course of ICP needs to be studied further.

This is the first study concerning the rationality of the classification of EOICP and LOICP. The study proved that the classification of EOICP and LOICP may be not unique and need to be reconsidered. But there are some limitations. First, we didn’t get the original data of Layer *et al*.’s study although we have tried every possible way to get the data. Thus, we were unable to present the accurate distribution with the accurate age at onset of ICP. However, we had tried our best to reduce the bias caused by data reconstruction. The mean value of lower and upper range of each age group in the bar graph (“Fig. 1” in Layer *et al*.’s article study^1^) was set as the reconstructed age of every patient in the same age group. The description of data showed few differences which indicated that the reconstruction process might not influence the result (Supplementary Table 6). Second, SPINK1 mutation wasn’t tested in our study. Thus, we failed to explore the relationship of SPINK1 mutation with ICP. Third, the retrospectively acquired data collected between January 2000 and December 2004 might introduce recall bias.

In conclusion, age at onset of ICP was proved to be right skewed distribution. Differences always exist in EOICP_a_ and LOICP_a_ no matter what the cut-off value is. From this perspective, EOICP and LOICP according to Layer *et al*.’s classification might be not unique and need to be reconsidered.

## Data Availability

Data will be available from the corresponding author on reasonable request.

## Supplemenatry information

Supplemenatry information.
