# Supplementary material for: Classification of Early-Onset and Late-Onset Idiopathic Chronic Pancreatitis Needs Reconsideration
Source: Sci Rep. 2020 Jun 26;10:10448. doi: 10.1038/s41598-020-67306-w (PMC7320187; doi:10.1038/s41598-020-67306-w)
Supplement: Supplementary file 1 — Supplemenatry information. [file 41598_2020_67306_MOESM1_ESM.pdf]

Supplementary Information

Classification of Early-Onset and Late-Onset Idiopathic Chronic Pancreatitis Needs Reconsideration

Short title: Classification of ICP Needs Reconsideration

Yu Liu[1*], M.D., Dan Wang[1*], M.D., Yi-Li Cai[2], M.D., Tao Zhang[2], M.D., Hua-Liang Chen[2], M.D., Lu Hao[3], M.D., Teng Wang[1] , M.D., Di Zhang[1], M.D., Huai-Yu Yang[1], M.D., Jia-Yi Ma[1], M.D., Juan Li[1], R.N., Ling-Ling Zhang[1], R.N., Cui Chen[1], R.N., Hong-Lei Guo[1], M.D., Ya-Wei Bi[1], M.D., Lei Xin[1], M.D., Xiang-Peng Zeng[1], M.D., Hui Chen[1], MD., Ting Xie[4], M.D., Zhuan Liao[1], M.D., Zhi-Jie Cong[5], M.D., Zhao-Shen Li[2], M.D., Liang-Hao Hu[1], M.D., Chinese Medical Doctor Association of Pancreatology.

[1] Department of Gastroenterology, Changhai Hospital, The Second Military Medical University, Shanghai, 200433, China; [2] Basic medical sciences, The Second Military Medical University, Shanghai, 200433, China;[3] Department of Gastroenterology, First Affiliated Hospital, Zhejiang University School of Medicine, Hangzhou, 310006, China; [4] Department of Gastroenterology, Zhongda Hospital, Southeast University, Nanjing, 210000, China; [5] Department of General Surgery, Renji Hospital, Shanghai Jiaotong University, Shanghai, 200120, China.

[*] ***Drs. Liu and Wang contributed equally to this study.***

Corresponding authors:

Liang-Hao Hu, M.D.

Department of Gastroenterology, Changhai Hospital

The Second Military Medical University

168 Changhai Road, Shanghai 200433, China

E-mail address: lianghao-hu@hotmail.com

Supplementary Figure legends

Supplementary figure 1. Probability plot for age at onset of ICP in our study. The probability plot (Chambers et al., 1983) is a graphical technique for assessing whether or not a data set follows a given distribution such as the normal or Weibull. The data are plotted against a theoretical distribution in such a way that the points should form approximately a straight line. Departures from this straight line indicate departures from the specified distribution. The straight red line in the middle of the graph was the fitted distribution line. The two solid outer red lines on the graph were the confidence interval for each percentile (rather than the entire distribution). The blue circle was the data of the study.

Supplementary figure 2. Empirical cumulative distribution function for age at onset of idiopathic chronic pancreatitis in our study. Description of empirical cumulative distribution function plot: In reliability analysis, many data sets consists of a set of failure times, which may be truncated at some limit value. The cumulative distribution function is defined as: $F(t) = prob(T < t)$, where T is the lifetime of a randomly selected unit. An empirical cumulative distribution function plot is a plot of the empirical cumulative distribution function versus failure time. The failure time is plotted on the horizontal axis. At each failure time, the following two points are calculated and plotted on the vertical axis: $y1= \frac{i-1}{n}$, $y2= \frac{i}{n}$, with n and i denoting the number of data points and the rank of the failure time, respectively. The last failure time only calculates one of these points. When all of the points are connected, a staircase type plot results. The vertical step is constant for the failure times, while the lengths of the horizontal steps

are determined by the distances between failure times. The red line was the fitted distribution line and the blue line was the data of the study.

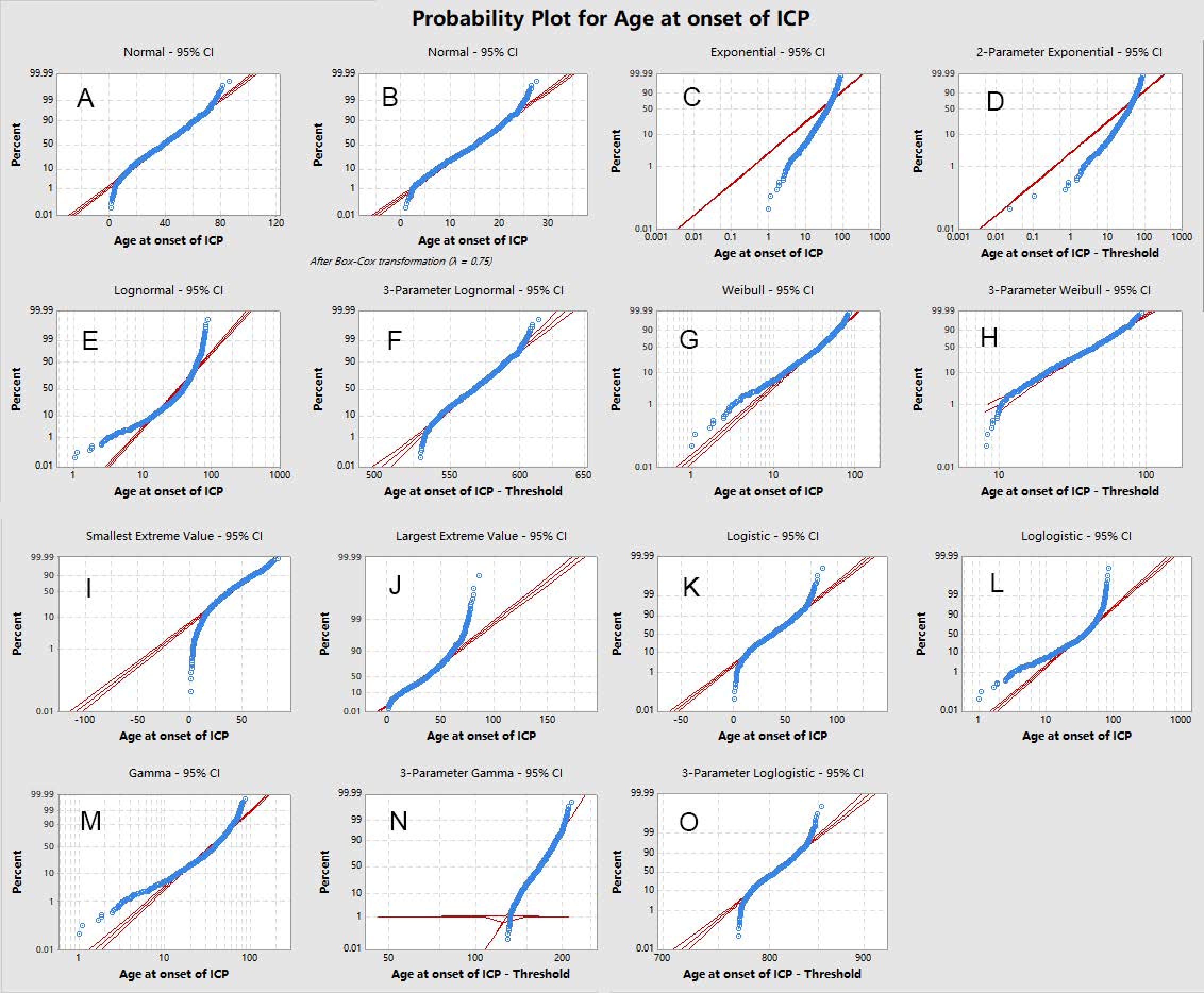

Probability Plot for Age at onset of ICP
Normal - 95% CI
A
Normal - 95% CI
B
After Box-Cox transformation (λ = 0.75)
Exponential - 95% CI
C
2-Parameter Exponential - 95% CI
D
Lognormal - 95% CI
E
3-Parameter Lognormal - 95% CI
F
Weibull - 95% CI
G
3-Parameter Weibull - 95% CI
H
Smallest Extreme Value - 95% CI
I
Largest Extreme Value - 95% CI
J
Logistic - 95% CI
K
Loglogistic - 95% CI
L
Gamma - 95% CI
M
3-Parameter Gamma - 95% CI
N
3-Parameter Loglogistic - 95% CI
O
Percent
Age at onset of ICP
Age at onset of ICP - Threshold

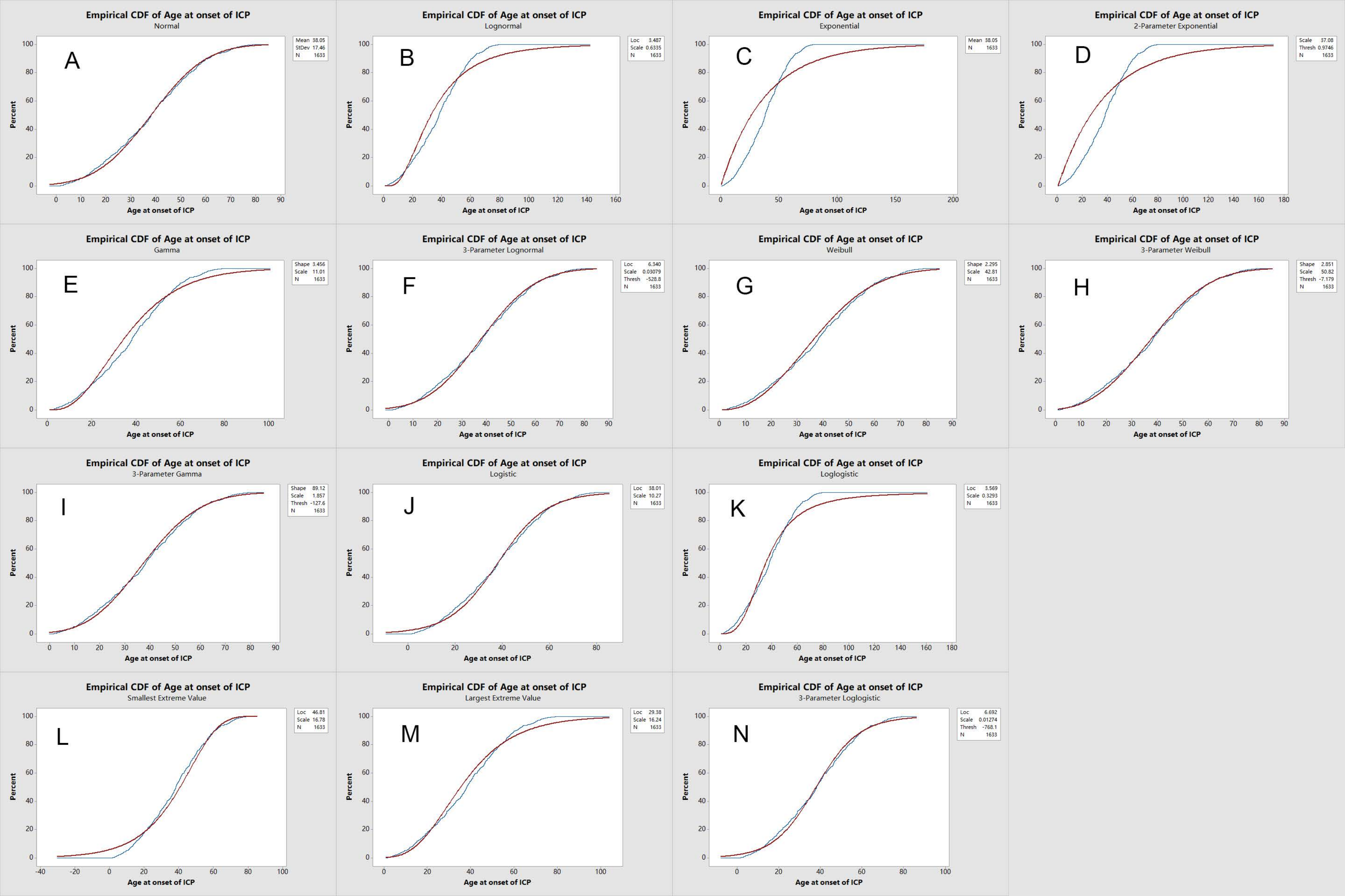

Empirical CDF of Age at onset of ICP
Normal
A
Mean 38.05
StDev 17.46
N 1633
Empirical CDF of Age at onset of ICP
Lognormal
B
Loc 3.487
Scale 0.6335
N 1633
Empirical CDF of Age at onset of ICP
Exponential
C
Mean 38.05
N 1633
Empirical CDF of Age at onset of ICP
2-Parameter Exponential
D
Scale 37.08
Thresh 0.9746
N 1633
Empirical CDF of Age at onset of ICP
Gamma
E
Shape 3.456
Scale 11.01
N 1633
Empirical CDF of Age at onset of ICP
3-Parameter Lognormal
F
Loc 6.340
Scale 0.03079
Thresh -528.8
N 1633
Empirical CDF of Age at onset of ICP
Weibull
G
Shape 2.295
Scale 42.81
N 1633
Empirical CDF of Age at onset of ICP
3-Parameter Weibull
H
Shape 2.851
Scale 50.82
Thresh -7.179
N 1633
Empirical CDF of Age at onset of ICP
3-Parameter Gamma
I
Shape 89.12
Scale 1.857
Thresh -127.6
N 1633
Empirical CDF of Age at onset of ICP
Logistic
J
Loc 38.01
Scale 10.27
N 1633
Empirical CDF of Age at onset of ICP
Loglogistic
K
Loc 3.569
Scale 0.3293
N 1633
Empirical CDF of Age at onset of ICP
Smallest Extreme Value
L
Loc 46.81
Scale 16.78
N 1633
Empirical CDF of Age at onset of ICP
Largest Extreme Value
M
Loc 29.38
Scale 16.24
N 1633
Empirical CDF of Age at onset of ICP
3-Parameter Loglogistic
N
Loc 6.692
Scale 0.01274
Thresh -768.1
N 1633
Percent
Age at onset of ICP

Supplementary Table 1. General characteristics of 1633 patients with idiopathic chronic pancreatitis.

Items	Number of patients	Percent (%)
Male sex	1031	63.1
Female sex	602	36.9
Age at the onset of CP, y*	38.054 (17.460)	
Age at the diagnosis of CP, y*	42.631 (16.431)	
Adolescent	248	15.2
Smoking history	372	22.8
Alcohol consumption		
0 g/day	1342	82.1
0~20 g/day	62	3.8
20~80 g/day or 60 g/day	229	14.0
Initial manifestations		
Abdominal pain	1346	82.4
Diabetes mellitus	178	10.9
Others	109	6.7
Pancreatic stones†	1192	73.0
Steatorrhea	339	20.8
DM	430	26.3
Biliary stricture	259	15.9
Pancreatic pseudocysts	240	14.7
Pancreatic cancer	18	1.1
Death	57	3.5
Morphology of MPD		
Pancreatic stone alone	455	27.9
MPD stenosis alone	497	30.4
MPD stenosis and stone	519	31.8
Complex pathologic changes	162	9.9
Type of pain		
Recurrent acute pancreatitis	487	29.8
Recurrent pain	514	31.5
Recurrent acute pancreatitis and pain	419	25.7
Chronic pain	79	4.8
Without pain	134	8.2
Severe acute pancreatitis	55	3.4
Pancreatic duct successful drainage‡	1119	68.5
Overall treatment		
Endotherapy alone	1105	67.7
Surgery alone	202	12.4
Both endotherapy and surgery	136	8.3
Conservative treatment	190	11.6

CP = chronic pancreatitis, DM = diabetes mellitus, MPD = main pancreatic duct

*Median (range).

†Pancreatic calcifications were also regarded as stones that are located in branch pancreatic duct or ductulus.

‡Patients with successful main pancreatic duct (MPD) drainage are those whose CP was established after endoscopic retrograde cholangiopancreatography or pancreatic surgery or those who underwent successful MPD drainage during administration when CP diagnosis was established.

Supplementary Table 2. Descriptive Statistics of distribution of age at onset of idiopathic chronic pancreatitis patients in Layer et al's study (n = 66) and in our population (n = 1,633).

Item	Mean	StDev	Median	Minimum	Maximum	Skewness	Kurtosis
Age at onset of ICP in our population, y	38.05	17.46	38.21	0.10	85.36	0.05 ± 0.06	2.33
Age at onset of ICP in Layer et al's population*, y	40.80	22.04	45.00	2.5	82.5	-0.175	-1.043

ICP = idiopathic chronic pancreatitis, StDev = standard deviation

* The "age at onset of ICP in Layer et al's study" was calculated by reconstructed data.

Supplementary Material

Supplementary Table 3. Goodness of Fit Test results of Individual Distribution Identification for age at onset of idiopathic chronic pancreatitis in our population (n = 1,633).

Distribution	AD	P	LRT P
Normal	2.988	<0.005	
Box-Cox Transformation	4.333	<0.005	
Lognormal	46.879	<0.005	
3-Parameter Lognormal	3.136	-	0.000
Exponential	195.276	<0.003	
2-Parameter Exponential	183.996	<0.010	0.000
Weibull	6.392	<0.010	
3-Parameter Weibull	2.559	<0.005	0.000
Smallest Extreme Value	15.658	<0.010	
Largest Extreme Value	14.655	<0.010	
Gamma	21.273	<0.005	
3-Parameter Gamma	3.632	-	0.000
Logistic	5.354	<0.005	
Loglogistic	27.827	<0.005	
3-Parameter Loglogistic	5.438	-	0.000

AD = Anderson-Darling goodness-of-fit statistic, P = probability, LRT = likelihood ratio test

Supplementary Material

Supplementary Table 4. Maximum Likelihood Estimates of Distribution Parameters for age at onset of idiopathic chronic pancreatitis in our population (n = 1,633).

Distribution	Location	Shape	Scale	Threshold
Normal*	38.05425		17.45990	
Box-Cox Transformation*	14.77248		5.35751	
Lognormal*	3.48740		0.63352	
3-Parameter Lognormal	6.33959		0.03079	-528.77899
Exponential			38.05425	
2-Parameter Exponential			37.07970	0.97455
Weibull		2.29524	42.81364	
3-Parameter Weibull		2.85082	50.81936	-7.17937
Smallest Extreme Value	46.80678		16.77913	
Largest Extreme Value	29.38201		16.23733	
Gamma		3.45570	11.01201	
3-Parameter Gamma		89.11868	1.85733	-127.55439
Logistic	38.00657		10.26561	
Loglogistic	3.56913		0.32934	
3-Parameter Loglogistic	6.69206		0.01274	-768.06986

* Scale: Adjusted maximum likelihood estimate

Supplementary Material

Supplementary Table 5. One-Sample Kolmogorov-Smirnov Test of age at onset of idiopathic chronic pancreatitis in Layer's study (calculated by reconstructed data, n=66).

	Age
N	66
Uniform parameters	
Minimum	2.5
Maximum	82.5
Most extreme differences	
Absolute	0.129
Positive	0.129
Negative	-0.076
Kolmogorov-Smirnov, Z	1.046
Asymptotic Significance (2-tailed)	0.224

Supplementary Material

Supplementary Table 6. Comparison description of age at onset of idiopathic chronic pancreatitis between original data in Layer et al's study and the reconstructed data.

Items	Layer et al's study	Reconstructed data
N	66	66
EOICP, n	25	25
LOICP, n	41	41
Median age		
EOICP (yr)	19.2	17.5
LOICP (yr)	56.2	57.5
